# Serum levels of bone sialoprotein correlate with portal pressure in patients with liver cirrhosis

**DOI:** 10.1371/journal.pone.0231701

**Published:** 2020-04-17

**Authors:** Fabian Benz, Andreas Bogen, Michael Praktiknjo, Christian Jansen, Carsten Meyer, Alexander Wree, Muenevver Demir, Sven Loosen, Mihael Vucur, Robert Schierwagen, Frank Tacke, Jonel Trebicka, Christoph Roderburg

**Affiliations:** 1 Department of Gastroenterology/Hepatology, Charité University Medicine Berlin, Berlin, Germany; 2 Department of Internal Medicine I, University Clinic Bonn, Bonn, Germany; 3 Department of Radiology, University Clinic Bonn, Bonn, Germany; 4 Department of Medicine III, University Hospital RWTH Aachen, Aachen, Germany; 5 Department of Internal Medicine I University Clinic Frankfurt, Frankfurt, Germany; 6 European Foundation for the Study of Chronic Liver Failure - EF CLIF, Barcelona, Spain; 7 Institute for Bioengineering of Catalonia, Barcelona, Spain; 8 Department of Gastroenterology and Hepatology, Odense University Hospital, Odense, Denmark; Medizinische Fakultat der RWTH Aachen, GERMANY

## Abstract

Liver cirrhosis represents the common end-stage of chronic liver diseases regardless of its etiology. Patients with compensated disease are mostly asymptomatic, however, progression to a decompensated disease stage is common. The available stratification strategies are often unsuitable to identify patients with a higher risk for disease progression and a limited prognosis. SIBLINGs, soluble glycophosphoproteins, are secreted into the blood by immune-cells. While osteopontin, the most prominent member of the SIBLINGs family, has been repeatedly associated with liver cirrhosis, data on the diagnostic and/or prognostic value of bone sialoprotein (BSP) are scarce and partly inconclusive. In this study, we analyzed the diagnostic and prognostic potential of circulating BSP in comparison to other standard laboratory markers in a large cohort of patients with liver cirrhosis receiving transjugular intrahepatic portosystemic shunt (TIPS). Serum levels of BSP were similar in patients with different disease stages and were not indicative for prognosis. Interestingly, BSP serum levels did correlate inversely with portal pressure, as well as its surrogates such as platelet count, the portal vein cross-sectional area and correlated positively with the portal venous velocity. In summary, our data highlight that BSP might represent a previously unrecognized marker for portal hypertension in patients with liver cirrhosis.

## Introduction

The natural course of liver cirrhosis, the common end-stage of all chronic liver diseases, consists of a compensated phase, which is asymptomatic in most patients, and a decompensated disease phase characterized by the occurrence of clinical complications, such as ascites or bleeding mainly driven by portal hypertension [1]. In this disease stage, selected patients benefit from a transjugular intrahepatic portosystemic shunt (TIPS) that decompresses the portal system by shunting an intrahepatic portal branch into a hepatic vein [1]. The transition from compensated to decompensated liver cirrhosis occurs at a rate of about 6% /year. Different markers and prognostic scores have been tested on their ability to stratify the risk of mortality in patients with compensated or decompensated liver cirrhosis as well as in patients with acute-on-chronic liver failure (ACLF). So far, biomarkers of systemic inflammation and infection have performed fairly well to distinguish patients with a stable disease course from those at higher risk of decompensation [2–4]. Moreover, biomarkers of macrophage activation and immune danger signals were demonstrated to predict outcome in patients with decompensated liver cirrhosis, especially in patients with alcoholic hepatitis [5].

Bone sialoprotein (BSP) is a novel glycophosphoprotein that plays a pivotal role in activating natural killer cells, neutrophils and macrophages [6]. BSP is a member of the Small integrin-binding ligand N-linked glycoproteins (SIBLINGs)-family that consists of five integrin-binding glycophosphoproteins (the bone sialoprotein (BSP), dentin matrix protein 1 (DMP1), dentin sialophosphoprotein (DSPP), matrix extracellular phosphoglycoprotein (MEPE) and osteopontin (OPN)). All members of the SIBLINGs family are soluble proteins that are secreted by different cells into the blood. SIBLINGs regulate a plethora of processes such as cell death, angiogenesis and ECM remodeling. In line, SIBLINGs have been proposed as biomarkers for different diseases including inflammatory and infectious diseases as well as cancers [7–14].

Elevated serum levels of OPN, another member of the SIBLINGs family, were recently analyzed in patients with liver cirrhosis. OPN levels positively correlated with total bilirubin, Model for End-Stage Liver Disease (MELD) score, MELD-Na score and monocyte count. Moreover, OPN was identified as an independent risk factor for mortality in ACLF and reflected macrophage activation in patients with liver cirrhosis and alcoholic hepatitis [5]. Since many analyses suggested comparable features of different SIBILINGs when tested on their function as a biomarker for a specific disease condition [15], we hypothesized that BSP might also represent a previously unrecognized marker in the context of liver cirrhosis. To address this question, we measured serum BSP levels in 99 patients with liver cirrhosis receiving TIPS. We specifically analyzed whether BSP levels might indicate an unfavorable disease course and outcome in patients with liver cirrhosis.

## Material and methods

### Study design, data collection and patient characteristics

As previously described [16, 17], 99 patients with liver cirrhosis and severe portal hypertension, selected for TIPS insertion, were enrolled at the Department of Internal Medicine I, University of Bonn, Germany into the study. General clinical characteristics are displayed in Table 1. Inclusion criteria were age between 18 and 80, cirrhosis caused by alcohol or viral hepatitis, and decompensated cirrhosis with indication for TIPS. Exclusion criteria were contraindications for TIPS placement, which were serum levels of bilirubin >5 mg/dL, spontaneous bacterial peritonitis, overt hepatic encephalopathy, pulmonary arterial hypertension and cardiac insufficiency as recently described [16, 17]. One to three weeks after TIPS insertion, an invasive control of the TIPS was performed as part of routine care. Biochemical blood analyses were performed using standard tests. The local ethics committee of the University of Bonn approved the study (029/13), and all patients agreed and signed an informed consent, in accordance with the Helsinki Declaration.

**Table 1:** Clinical and laboratory parameters of all patients (n = 99).

Parameters	
Gender (male/female) [%]	67 / 33
Age [in years]	59 (36–77)
BMI [kg/m^2^]	24.5 (15.2–38.9)
Aetiology (alcoholic / non-alcoholic) [%]	73 / 27
MELD score	10 (6–33)
Child category (A / B / C) [%]	17 / 66 / 16
Ascites (no / mild / severe) [%]	19 / 17 / 64
Hepatic encephalopathy (no / yes) [%]	85 / 15
Hepatorenal syndrome Type 1 (no / yes) [%]	77 / 23
Oesophageal varices (no / grade I-II / grade III-IV) [%]	12 / 66 / 22
Sodium [mmol/l]	135 (119–143)
Potassium [mmol/l]	4.2 (2.6–5.8)
Creatinine [mg/dl]	1.1 (0.5–8.2)
Urea [mg/dl]	44 (9–225)
Bilirubine [mg/dl]	1.3 (0.4–16.9)
Albumine [g/dl]	32 (11–56)
INR	1.13 (0.95–2.40)
Thrombocytes [/nl]	104 (27–389)
Leukocytes [/nl]	5.5 (1.4–22.3)
GOT [U/l]	21 (8–73)
GPT [U/l]	18 (4–113)
GGT [U/l]	57 (8–1469)
BSP (HV) [ng/ml]	3.0 (0.0–134.2)
BSP (PV) [ng/ml]	4.9 (0.0–84.9)

Data are shown as median and ranges or percent of patients

### Transjugular intrahepatic portosystemic shunt insertion and haemodynamic measurements

TIPS (8–10 mm Viatorr Gore Medical, USA) placement was performed as previously described [16–18]. A single shot of antibiotic prophylaxis of cefuroxime (1.5 g) was administered at TIPS placement. Portal and hepatic venous pressures were measured invasively using a pressure transducer system (Combitrans, Braun Melsung, Germany) and a multichannel monitor (Sirecust, Siemens, Germany). The difference between portal and hepatic venous pressures was defined as portal hepatic pressure gradient (PHPG). The arterial pressure and heart rate were monitored noninvasively. Biochemical parameters as well as portal and systemic haemodynamics were recorded. The blood from the portal and the hepatic vein was collected as previously described [16–18]. The blood sample from the portal vein was taken immediately after puncture of the vein. The hepatic venous sample was taken from the hepatic vein, which was used for the creation of the TIPS, right before puncturing portal vein. Immediately after entering the portal vein, but before dilation of the tract or insertion of the TIPS-stent portal venous samples were taken. At invasive TIPS control after a median of 10 days range (1–3 weeks), the catheter was advanced into the portal vein. Blood from the portal and hepatic vein were collected.

### Determination of bone sialoprotein serum concentrations by ELISA

BSP serum concentrations were analyzed using a commercial enzyme immunoassay according to the manufacturers’ instructions (Human BSP ELISA, Abbexxa, Cambridge, United Kingdome, No. abx575181).

### Statistical analysis

All statistical analyses were performed with SPSS (SPSS, Chicago, IL, USA) or GraphPad Prism 5.0 as previously described [19]. Data are presented as mean ± standard error (SEM) or medians and ranges. The Wilcoxon test was used for comparison of paired data and the Mann-Whitney and Kruskal-Wallis test for unpaired comparisons. The box of box plots represents the interquartile range (IQR, 25th percentile to 75th percentile) with median (50th percentile) inside. Whiskers are drawn up to the largest observed point from the dataset with a distance of 1.5 times the IQR. Outliers are marked as small circles and "extreme values" are marked with a star. Correlations were analyzed with the Spearman correlation coefficient. Kaplan-Meier curves were used to analyze the survival rates of patients using the log-rank test. Cox regression multivariate analysis (forward stepwise likelihood-quotient) was performed to predict survival rates using the log-rank test. p values <0.05 were considered statistically significant.

## Results

### General patient characteristics

In this study, a total of 99 patients with decompensated cirrhosis (33% female, 67% male) were included. The median age at TIPS procedure was 59 (36–77) years. Alcohol was the most common etiology of liver cirrhosis (73%). The median MELD score was 10 (6–33). Before TIPS implantation, most patients had esophageal varices (III° in 66%; IIIIV° in 22%) and ascites (mild 17%, severe 64%). Characteristics of the study population are summarized in Table 1.

### BSP serum levels correlate with portal pressure and its surrogates in patients with liver cirrhosis

**Fig 1:**
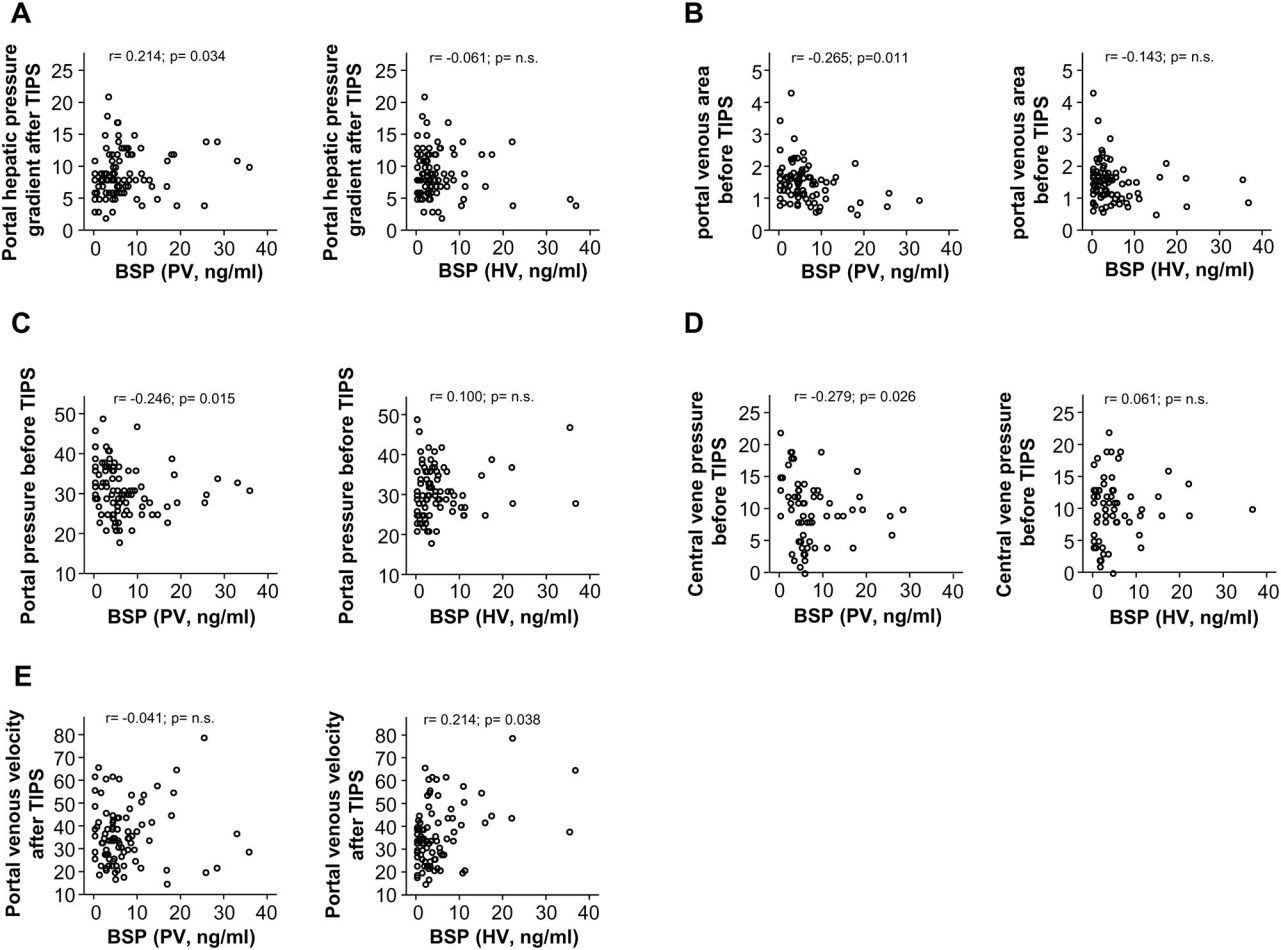
Serum levels of BSP significantly correlated to markers for portal pressure. BSP serum levels were correlated to different laboratory parameters routinely assessed in patients with liver cirrhosis as well as with markers for portal pressure including portal pressure before TIPS, portal hepatic pressure gradient after TIPS and portal venous velocity after TIPS.

In order to evaluate a potential role of BSP serum concentrations as a biomarker in the context of patients with liver cirrhosis and patients receiving transjugular intrahepatic portosystemic shunt we first performed Spearman`s correlation analysis between BSP serum levels and parameters indicating portal pressure. Strikingly, BSP levels were significantly correlated with the portal pressure before TIPS (PV: r = -0.246; p = 0.015; HV: 0100, p = n.s), as well as its surrogates such as portal venous velocity (PV: r = -0.041, p = n.s.; HV: r = 0.214; p = 0.038) and portal vein crosssectional area (HV: r = -0143, p = n.s.; PV: r = -0.265; p = 0.011). Moreover, serum concentrations of BSP were significantly correlated with an impaired kidney function (Creatinine PV: r = 0.130, p = n.s; HV: r = 0.292, p = 0.003) as well as thrombocyte count (PV: r = 0.307, p = 0.002; HV: r = -0.058, p = n.s.; Fig 1).

In contrast, BSP levels were not correlated with serum levels of sodium, potassium, albumin (S1 Fig), urea, bilirubin, aspartate/ alanine-aminotransferases, cholinesterase, gamma-glutamyltransferase, INR, ammonium and markers of systemic inflammation or infection. Moreover, serum BSP concentrations were independent on patients´ BMI (S2 Fig).

### BSP serum levels are not elevated in patients with decompensated liver cirrhosis

**Fig 2:**
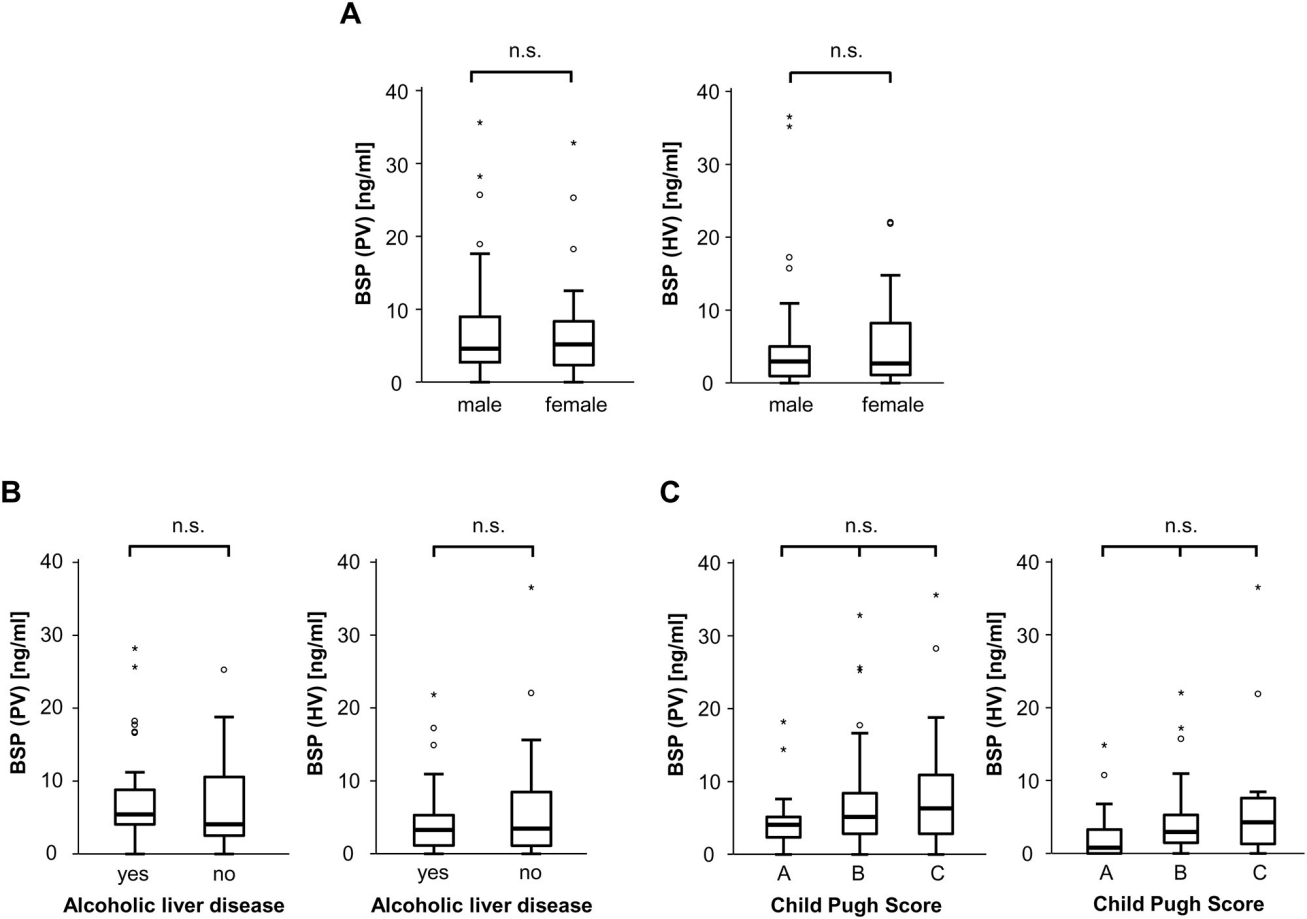
Serum levels of BSP in patients with liver cirrhosis. (A) BSP serum levels (both portal-venous as well as from liver vein) in cirrhotics did not differ between male and female patients. (B, C) BSP serum levels (both portal-venous (PV) as well as from liver vein (HV)) were independent from the disease etiology (alcoholic or not) and the disease stage (according to the Child-Pugh class) in patients with liver cirrhosis.

Based on these striking results, we next analyzed serum levels of BSP (both portal-venous (PV) as well as from liver vein (HV)) in a large and well-characterized cohort of patients with liver cirrhosis receiving TIPS (Table 1). To analyze the impact of different patients´ characteristics on BSP levels within our cohort, patients were subdivided by sex (Fig 2A), alcoholic disease etiology (Fig 2B) and Child classes (Fig 2C), and BSP levels were analyzed within the different subgroups of patients. However, there were no significant differences between these subgroups (Fig 2A–2C).

**Fig 3:**
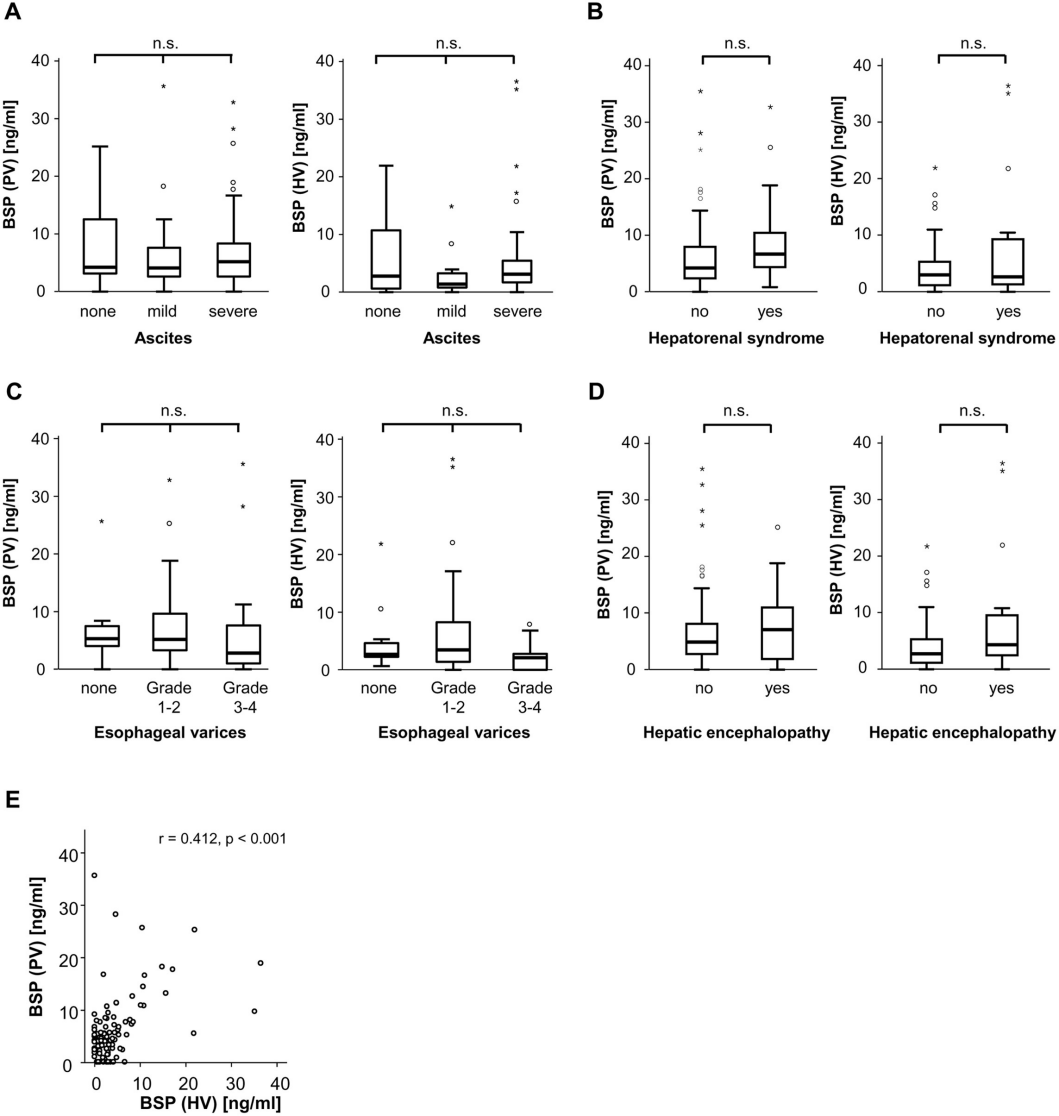
Serum levels of BSP in different subgroups of patients with liver cirrhosis. (A) BSP serum levels (both portal-venous as well as from liver vein) were independent on the presence of ascites in patients with liver cirrhosis. (B) BSP serum levels (both portal-venous (PV) as well as from liver vein (HV)) were similar in patients with or without hepatorenal syndrome. (C) The presence of esophageal varices had no influence on BSP serum concentrations (both portal-venous (PV) as well as from liver vein (HV)) in patients with liver cirrhosis. (D) BSP serum levels (both portal-venous as well as from liver vein) were unaltered between cirrhotics with or without hepatic encephalopathy. (E) BSP serum levels from portal-venous and liver vein demonstrated an almost perfect correlation.

Next, we compared concentrations of BSP between patients with different complications of disease. However, BSP levels did not vary between patients with or without ascites (Fig 3A), hepatorenal syndrome or normal kidney function (Fig 3B), presence or absence of esophageal varices (Fig 3) and with or without hepatic encephalopathy (Fig 3D).

Notably, in these analyses, BSP concentrations were very similar in samples from liver and portal veins and BSP serum levels were strongly correlated to each other (Fig 3E).

### No association between BSP serum concentrations with patients’ survival

**Fig 4:**
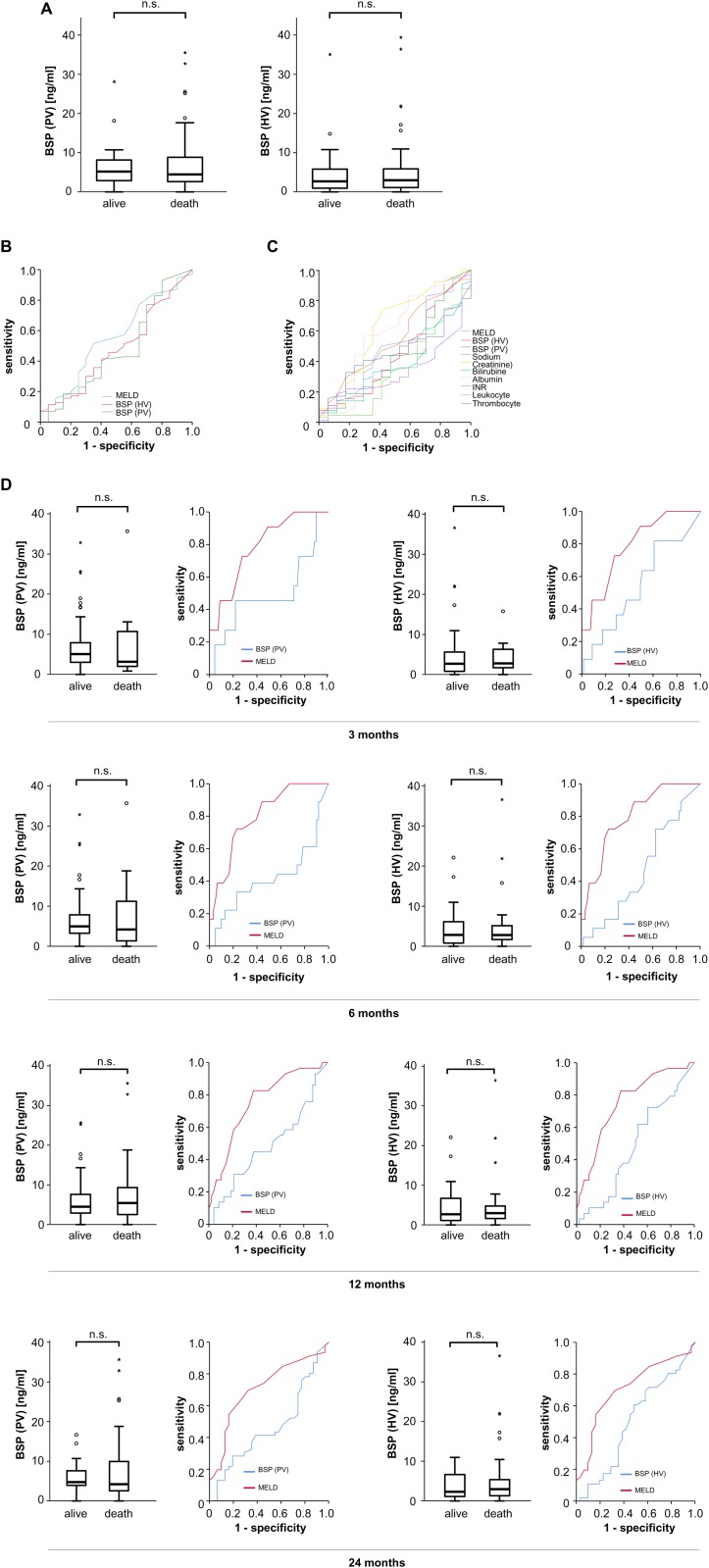
Association of BSP serum concentrations and patients survival. (A) BSP serum levels were similar in patients that survived compared to patients that died from liver cirrhosis during follow-up. (B) ROC curve analysis showed that the prognostic value of BSP serum levels in patients with liver cirrhosis is poor (C) ROC curve analysis showed that serum levels of BSP were inferior to other scoring systems or serum markers in differentiating between patients that survived or not. (D) SBP serum levels were similar in patients that survived and patients that died from liver cirrhosis at different time-points (left panels); ROC curve analysis showed that serum levels of BSP had an inferior prognostic value compared to the MELD score in patients with liver cirrhosis.

Serum levels of SIBLINGs were recently identified as prognostic markers for different diseases [7–9]. Based on these data, we next aimed at analyzing whether BSP concentrations are suitable to predict mortality in patients with liver cirrhosis. Therefore, we first examined BSP serum concentrations in cirrhotic patients that succumbed to death and those patients who survived. Interestingly, BSP levels did not vary with respect to patients’ survival (Fig 4A) and displayed an only very modest value in discriminating between survivors and deceased patients during follow-up (Fig 4B), especially when compared to other routinely assessed markers (Fig 4C).

We next performed Kaplan Meier analysis to determine the impact of BSP serum concentrations on patients´ survival in our cohort of patients. Despite using different cut-off values (median BSP serum levels, lower/ upper quartile of BSP serum concentrations), these analyses revealed that patients with higher vs lower BSP levels (both portal-venous as well as from liver vein) had an almost identical prognosis (S3 Fig, Table 2).

**Table 2:** AUC values of different markers for prediction of overall survival.

Marker	AUC
BSP (PV)	0.441
BSP (HV)	0.456
MELD	0.506
Sodium	0.547
Bilirubine	0.391
Creatinine	0.627
Albumine	0.589
INR	0.334
Leukocytes	0.349
Thrombocytes	0.460

Finally, we hypothesized that the prognostic role of BSP concentrations might be time dependent. Therefore, we subdivided our cohort of patients into those that had died or were alive 3, 6, 12 and 24 months after TIPS and analyzed BSP serum levels in the different sub-groups. However, also in these analyses, no differences became apparent and ROC curve analysis revealed that BSP had no prognostic power to estimate survival of patients with liver cirrhosis 3, 6, 12 and 24 months (Fig 4D, Table 3, S4 Fig).

**Table 3:** AUC values of different markers for prediction of patients´ survival at different time points.

	AUC of BSP (PV)	AUC of BSP (HV)	AUC of MELD
**3 months**	0.497	0.547	0.789
**6 months**	0.418	0.472	0.797
**12 months**	0.485	0.486	0.754
**24 months**	0.464	0.501	0.710

## Discussion

In this study, we measured serum concentrations of BSP in a large and well-characterized cohort of 99 patients with liver cirrhosis receiving TIPS. We observed that serum levels of BSP were similar in patients with different diseases stages and were not associated with patients´ outcome. In contrast, BSP serum levels were correlated with portal pressure and surrogates of portal hypertension. While these data argue against a role of BSP as a prognostic biomarker in patients with liver cirrhosis, they highlight that BSP may be helpful in the non-invasive diagnosis of portal hypertension. Moreover, this work underscores the previously suggested complex role of the SIBLINGs family in the pathophysiology of cirrhosis (e.g. [20, 21]).

Portal hypertension is the main driver of complications in liver cirrhosis, especially development of ascites and variceal bleeding. Both complications are successfully treatable by portal pressure lowering strategy using TIPS in selected patients. It has been clearly shown that TIPS improves survival in these patients and thereby changes the natural history of the disease. Another advantage of TIPS was used in this study, namely the invasive and direct measurement of portal pressure. Usually the portal pressure is measured invasively using hepatic venous pressure gradient (HVPG). Indeed, HVPG is an excellent indicative of outcome in patients with liver cirrhosis and portal hypertension. The clinically significant portal hypertension is defined as HVPG above 10mmHg and is associated with development of complications and decompensation episodes. Our study demonstrated that BSP may be a surrogate of portal hypertension as shown by the inverse correlation with portal pressure. Moreover, further lines of evidence suggest that this is probably. First the cross-sectional area of the portal vein is higher suggesting portal venous congestion in line with portal hypertension, second lower platelet counts are also associated with portal hypertension, and finally the lower portal vein velocity also is surrogate of portal hypertension. All these parameters were correlated with BSP levels and thereby underline a potential role of BSP as a biomarker of portal hypertension. Although all our patients had clinically significant portal hypertension treated by TIPS, there was still a statistically significant correlation of the lower BSP levels with higher portal pressure levels, which urges for confirmation and validation in a larger population with a wider range of portal pressure.

This finding is novel. Especially, since BSP has been related so far to inflammation. BSP is a member of the SIBLING (Small Integrin-Binding Ligand, N-linked Glycoprotein) family of genetically related proteins that are clustered on human chromosome 4 [9, 15, 22]. These proteins, initially identified in bone and teeth, share many structural characteristics [9, 15, 22] and play a critical role in various inflammatory diseases. As such, we have demonstrated that circulating levels of both OPN, the probably most prominent member of the SIBLINGs family, and BSP are elevated in patients with critical illness and sepsis [7, 14]. Moreover, both proteins were associated with an unfavorable disease course and correlated with lower survival. Important roles for SIBLINGs have further been suggested in various immune mediated diseases (multiple sclerosis, rheumatoid arthritis, lupus and related diseases, Sjögren’s disease, colitis [23] and cancers [9, 10, 24], suggesting a role for SIBLINGs in diseases associated with an systemic inflammatory response. In the past OPN was identified as a novel diagnostic and prognostic marker in the context of liver cirrhosis [20] and OPN serum levels were associated with the degree of liver injury and development of clinical complications in these patients. Notably, in our recent analysis in critical illness and cancer, elevated levels of OPN and BSP correlated with routinely used markers of liver injury and an impaired liver synthesis capacity [7, 9, 10, 14]). These previous reports suggest a direct role of BSP in the pathophysiology of liver cirrhosis (summarized in [21]). However, we could not confirm that BSP was associated with outcome. This may be explained by several reasons. On the one hand the BSP levels were measured in samples acquired at TIPS insertion and TIPS changes the natural history. Therefore, the BSP levels may not predict outcome. On the other hand BSP was correlated with portal pressure and TIPS decreases portal pressure and thereby again decreases the risk for complications of cirrhosis. Probably in another cohort of patients prognostic value of BSP may play a more obvious role.

Our study faces several important limitations. The first limitation is due to the selection of patients, in which all patients received TIPS and presented with portal hypertension and were previously decompensated. Therefore, a deep insight in the natural history of disease is missing in this context. Moreover, some of the analyzed subgroups were relatively small in terms of patients´ numbers in each group. Finally, the retrospective evaluation of the samples and the missing longitudinal approach may also induce a bias. Therefore, larger prospective studies are needed to finally state about the role of circulating BSP as a marker in patients with liver cirrhosis, a group of patients with a still unacceptably poor prognosis.

## Conclusion

In summary, our data argue against a general role for members of the SIBLINGs family in diagnosis or the prognostic judgment of patients with liver cirrhosis. Circulating BSP correlated with portal pressure and its surrogates and may represent one of the few available non-invasive markers for portal hypertension. Of course, these data need to be confirmed in further longitudinal clinical trials using independent cohorts. However if these data can be confirmed, our results might open the door for a potential clinical use of circulating BSP for non-invasive diagnosis of portal hypertension. Moreover, our results underscore the complexity of the regulation of SIBLINGs in liver cirrhosis and should trigger further mechanistic research on the role of BSP and SIBLING proteins in this context.

## Supporting information

S1 Fig Association of BSP serum concentrations and patients´ albumin serum concentrations. Serum levels of BSP were independent of patients´ albumin serum concentrations.
(PDF)

S2 Fig Association of BSP serum concentrations and patients´ BMI. Serum levels of BSP were independent of patients´ BMI.
(PDF)

S3 Fig Correlation of central-venous and portal-venous BSP concentrations in patients with liver cirrhosis. Kaplan Meier curve analysis using different cut-offs showed that BSP serum levels did not reflect survival of patients with liver cirrhosis.
(PDF)

S4 Fig Association of BSP serum concentrations and patients survival. ROC Analysis comparing the value of BSP levels to discriminate between survivors and patients that died at the indicated time points. Liver vein and portal-venous BSP have an almost identical value in discriminating between survivors and patients that succumbed to death.
(PDF)
