# Supplementary figures and images for: Serum levels of bone sialoprotein correlate with portal pressure in patients with liver cirrhosis
Source: PLoS One. 2020 Apr 17;15(4):e0231701. doi: 10.1371/journal.pone.0231701 (PMC7164617; doi:10.1371/journal.pone.0231701)

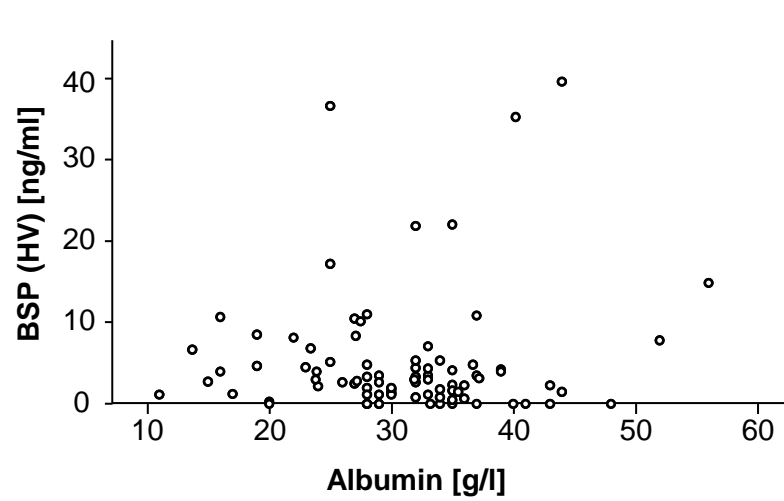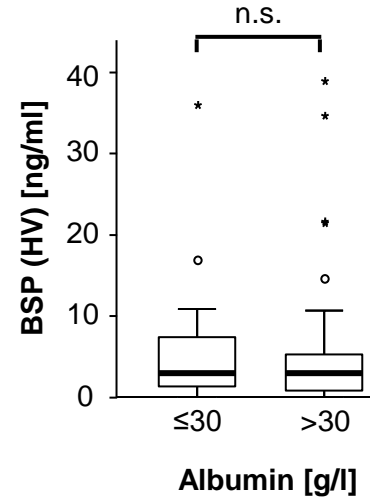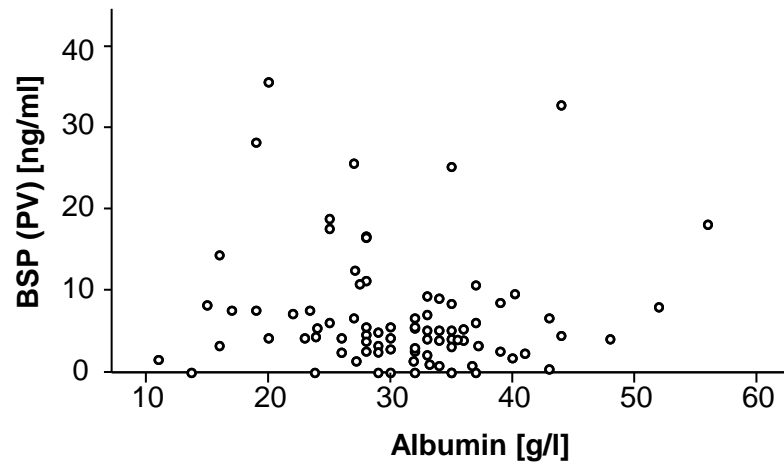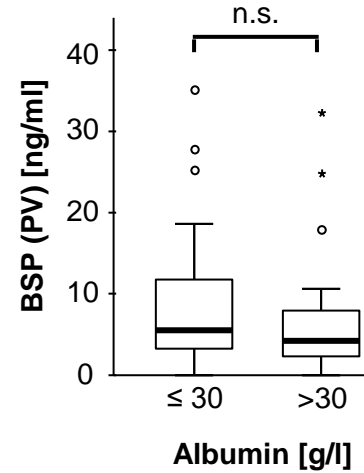

Supplement: S1 Fig — Serum levels of BSP were independent of patients´ albumin serum concentrations. (PDF) [file pone.0231701.s001.pdf]

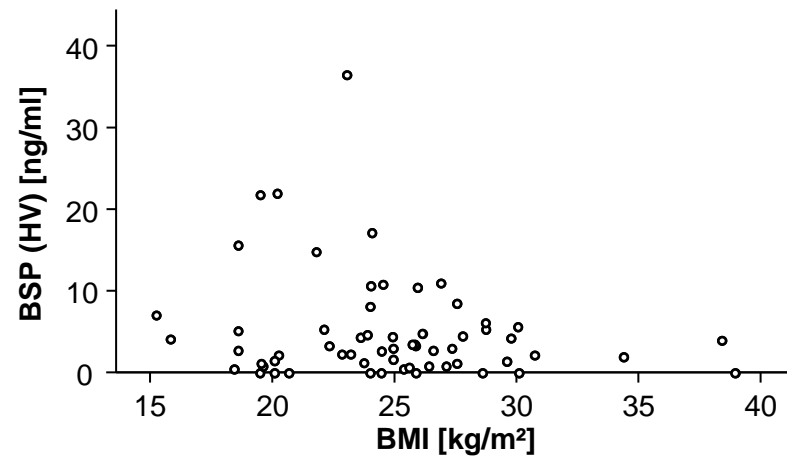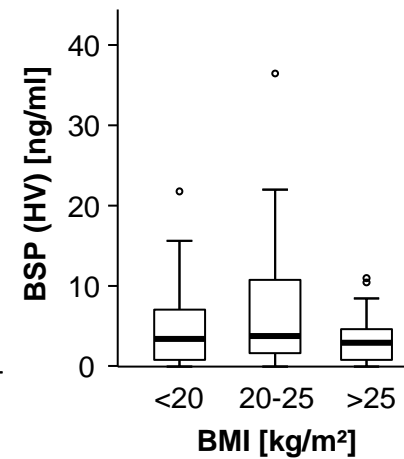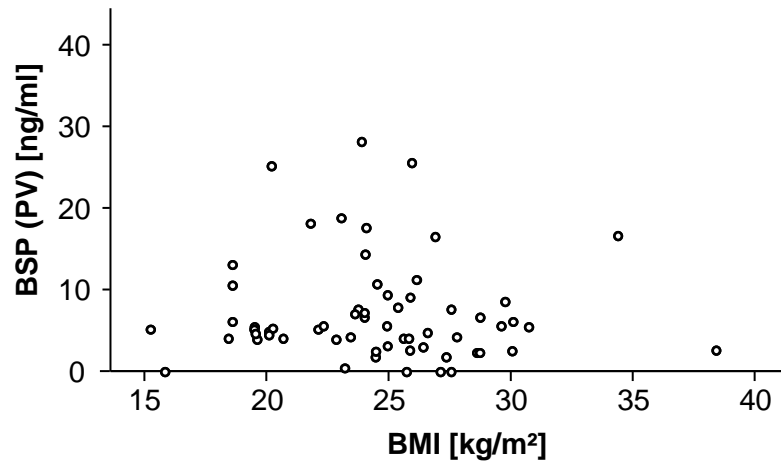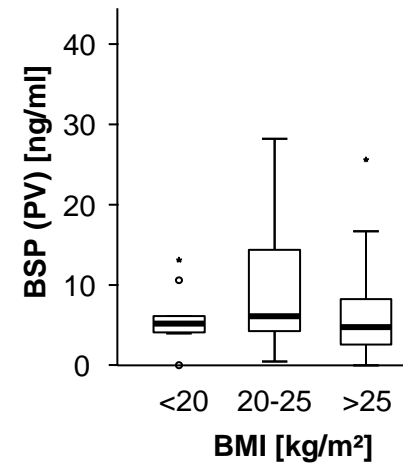

Supplement: S2 Fig — Serum levels of BSP were independent of patients´ BMI. (PDF) [file pone.0231701.s002.pdf]

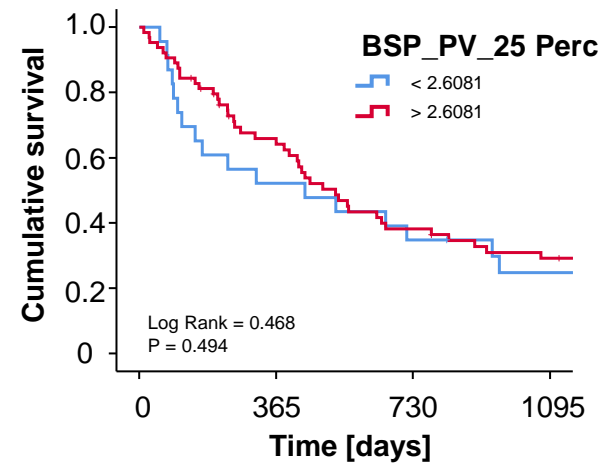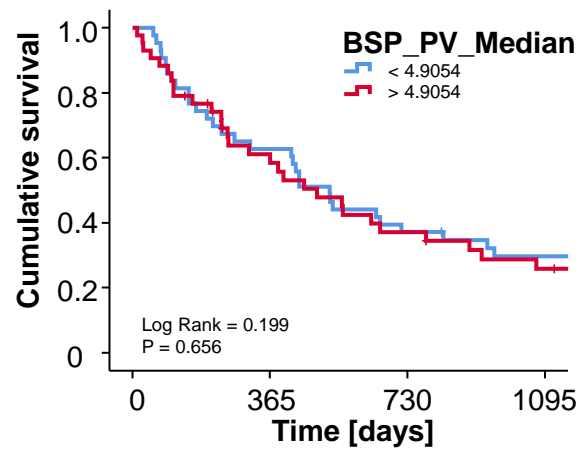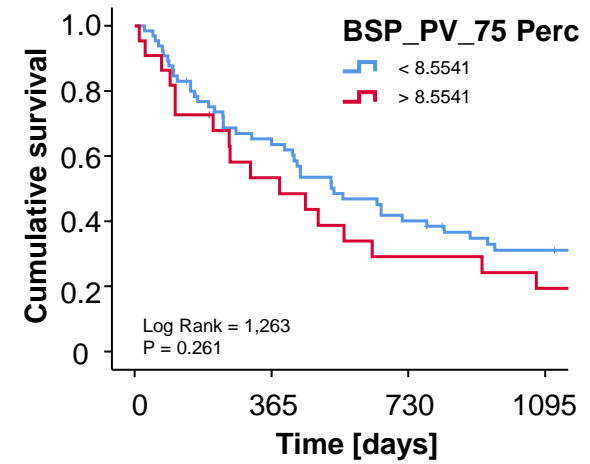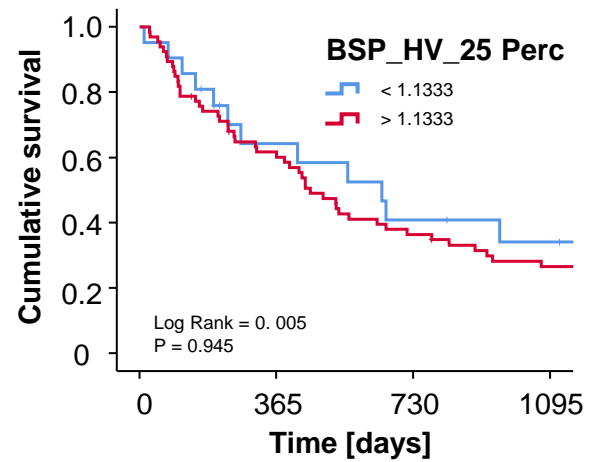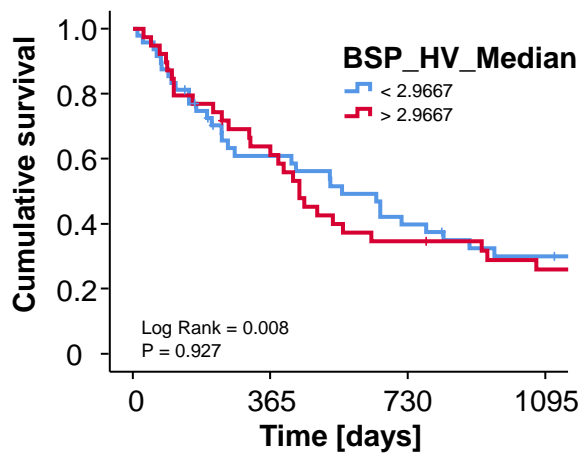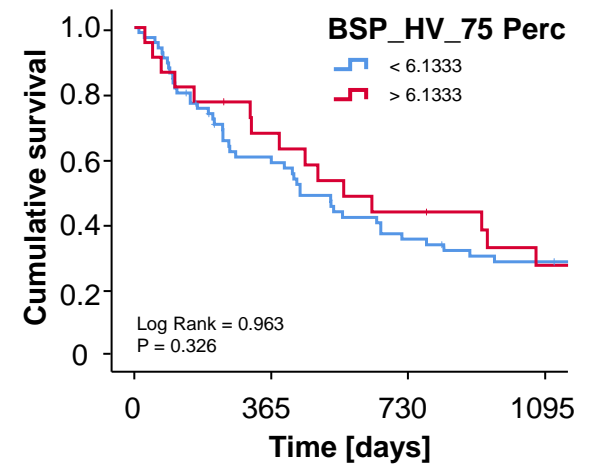

Supplement: S3 Fig — Kaplan Meier curve analysis using different cut-offs showed that BSP serum levels did not reflect survival of patients with liver cirrhosis. (PDF) [file pone.0231701.s003.pdf]

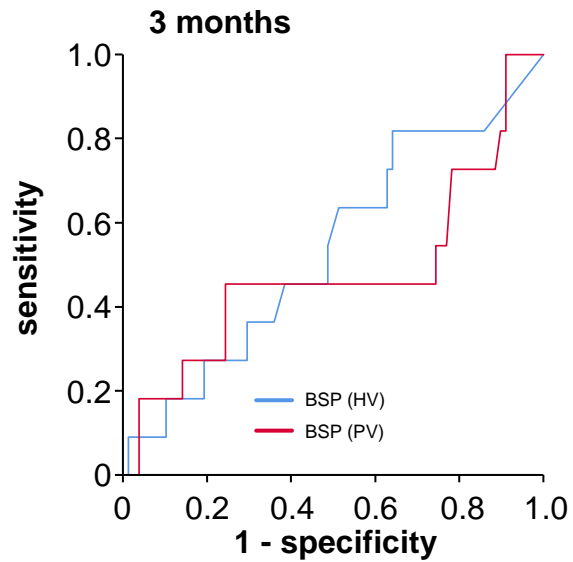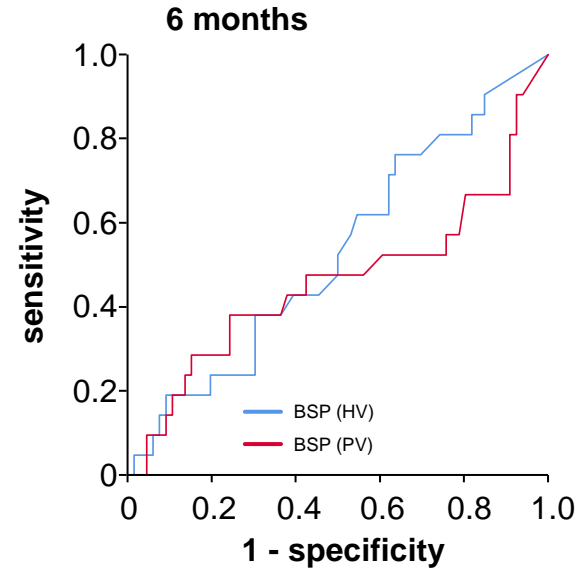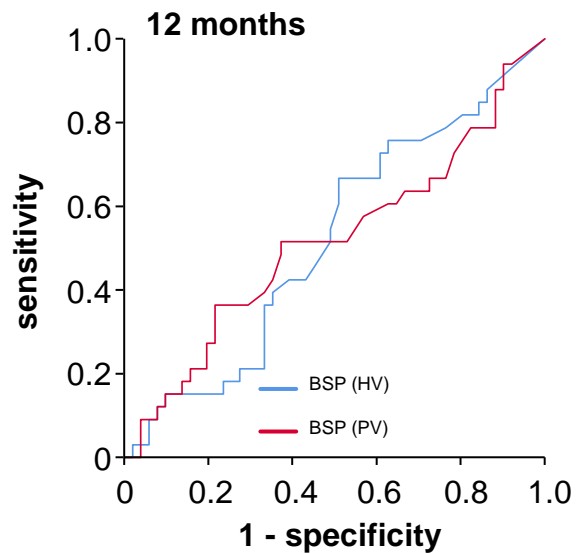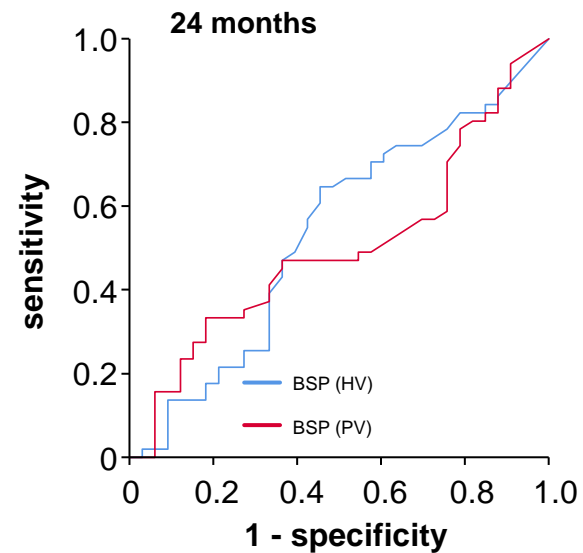

Supplement: S4 Fig — ROC Analysis comparing the value of BSP levels to discriminate between survivors and patients that died at the indicated time points. Liver vein and portal-venous BSP have an almost identical value in discriminating between survivors and patients that succumbed to death. (PDF) [file pone.0231701.s004.pdf]
